# New Insights into ZIF-90 Synthesis

**DOI:** 10.3390/molecules29163731

**Published:** 2024-08-06

**Authors:** Jan Marčec, Alenka Ristić, Nataša Zabukovec Logar

**Affiliations:** 1 National Institute of Chemistry, Hajdrihova 19, SI-1000 Ljubljana, Slovenia; jan.marcec@ki.si (J.M.); natasa.zabukovec@ki.si (N.Z.L.); 2 School of Science, University of Nova Gorica, Vipavska cesta 13, SI-5000 Nova Gorica, Slovenia

**Keywords:** ZIF-90, solvothermal synthesis, microwave synthesis, water adsorption, hydrothermal stability

## Abstract

Zeolitic imidazolate frameworks (ZIFs) are traditionally synthesized using N, N-dimethylformamide (DMF). However, DMF is toxic and hazardous to human health and the environment, hence other alternative solvents need to be considered. Herein, three different solvents like methanol, water and acetone were used to replace DMF and to explore the syntheses of ZIF-90 using a conventional and a microwave-assisted solvothermal method to obtain hydrothermally stable products, which also exhibit an increased water uptake. Pure ZIF-90 was synthesized under ambient pressure at 60 °C for 90 min using the conventional solvothermal method in an acetone–water solution, while under microwave irradiation it was formed in only 5 min at 80 °C. Altering methanol, water and acetone in the reaction mixture significantly affected the structural and water adsorption properties of ZIF-90s, which were monitored via PXRD, TGA, nitrogen and water sorption, and SEM. The highly efficient, less toxic, low-cost and activation-free microwave synthesis resulted in the formation of ZIF-90 nanoparticles that exhibited the highest maximum water adsorption capacity (0.37 g/g) and the best hydrothermal stability between water adsorption at 30 °C and desorption at 100 °C at 12.5 mbar among all the products obtained.

## 1. Introduction

Zeolitic imidazolate framework materials (ZIFs) are a subclass of metal organic framework materials. They are usually synthesized by the reaction of hydrated transition metal salts (Zn, Co, etc.) with imidazolate linkers, resulting in the formation of topologies resembling zeolites, e.g., SOD, LTA, RHO, etc. By combining different imidazolate derivatives and tetrahedral metal nodes, a wide range of structures with good chemical and thermal stability, permanent porosity and large surface area can be formed [1]. ZIF-90, as one of the most studied ZIFs for the adsorption of various gasses [2,3,4,5,6], has a sodalite topology (SOD) with a pore entrance of 0.35 nm and a pore/cage diameter of 1.12 nm [7]. Several syntheses of ZIF-90 have been reported (Table 1) [2,3,7,8,9,10]. However, many of these methods required the use of N,N-dimethylformamide (DMF) as a solvent, which is considered toxic and not environmentally friendly, hence other suitable solvents need to be considered [11]. Škrjanc et al. [10] successfully synthesized ZIF-90 using two bio-based solvents, Cyrene™ and γ-valerolactone. The authors showed that the material prepared in Cyrene™ exhibited lower porosity after activation, whereas when prepared in γ-valerolactone, both crystallinity and porosity were retained after activation. Liu et al. [12] prepared superhydrophobic ZIF-90 in a methanol solution for the recovery of bioalcohols. The hydrophobic property was post-synthetically modulated with pentalfluorobenzylamine. The superhydrophobic ZIF-90 was able to recover 98% of bioalcohols from the mixture of bioalcohols and water. Shieh et al. [13] synthesized ZIF-90 using a water-based synthesis with the addition of polyvinylpyrrolidone (PVP) polymer. Using this approach, they obtained micron-sized particles, while nanoparticles were obtained by adding alcohol to the solvent mixture. Although this approach is attractive due to the principles of green chemistry, the specific surface area was lower than that of ZIF-90 prepared with DMF. As a part of the EU’s Green Deal, particularly under the Chemicals Strategy for Sustainability, it is essential to eliminate harmful substances and identify sustainable alternatives. The use and sale of DMF is severely restricted in the EU from 2023 [14,15]. While the use of water as a replacement for DMF would be preferable, as it is the most environmentally friendly and available solvent, the poor solubility of 1H-imidazole-2-carbaldehyde linker in water and the need to add a base to deprotonate it, requires further search for alternative less toxic dipolar aprotic solvents to replace DMF.

Microwave-assisted synthesis (MAS) is a commonly used technique in the field of inorganic and organic chemistry. It provides a route for obtaining compounds with narrow particle size distribution, short crystallization time, easy morphology control, etc., [21]. In addition, the introduction of microwave-assisted synthesis in the field of MOF is advantageous because (i) the mixture is heated rapidly, (ii) controlled cooling, (iii) particle size and composition are uniform, and (iv) little energy is consumed [22]. Several MAS of ZIFs have been proposed using different solvents, for example Butova et al. [23] synthesized ZIF-65 with micro- and mesopores for hydrogen storage in 15 min using DMF. They found that microwave synthesis has a beneficial effect on the material properties, as a hierarchical structure, which has a higher hydrogen storage capacity than the ZIF-65 obtained by a conventional synthesis method. ZIF-8 with different structural properties was prepared by different authors using MAS [24,25,26,27]. Xing et al. [24] produced ZIF-8 using the non-ionic triblock copolymers P123 and F127. Among other things, they investigated the influence of synthesis time on the formation of nanoparticles. They proved that the synthesis of ZIF-8 with a high specific surface area was possible in only one minute. Lai et al. [28] synthesized ZIF-8 within 30 min at 120 °C using methanol as a solvent. They found that the reaction temperature and pressure had a great influence on the formation of ZIF-8. In addition, the concentration of reactants affected the particle size and crystallinity of the material. Lucero et al. [29] synthesized micrometer-sized ZIF-11 with a specific surface area of 713 and 745 m^2^/g in 15 and 60 min, respectively.

For the use of the material in different applications, hydrothermal stability is a crucial property that needs to be investigated. Since ZIF-90 exhibits hydrophilic properties due to the carbaldehyde groups in the structure, it can be suitable in water adsorption applications, especially in the field of adsorption-based thermal batteries [30], where heat is stored when it is not needed, and is allowed to be released on users’ demand [31]. Adsorption thermal battery is based on reversible adsorption or discharging process (exothermic phenomenon) and desorption or charging process (endothermic phenomenon) between the adsorbents and the adsorbates. The efficiency of this adsorption technology is determined by the performance of the used adsorbent, which strongly depends on the structural properties correlated with the synthesis procedures. In the last decade, a growing interest in the adsorption thermal battery and subsequent rapid development of this technology has been observed [32]. Specifically, great efforts have been made in the research of adsorbent materials for better adsorption properties. Thus, the adsorbents should have a high adsorption capacity and structural stability during several sorption processes, a low desorption (charging) temperature and non-toxic, environmentally friendly and non-flammable characteristics. Several groups investigated the water adsorption properties of ZIF-90 [3,8,33]. Gao et al. [8] explained the hydrophilicity of the prepared ZIF-90 based on the isotherm and heat of adsorption, which was then compared with computer simulations. The role of van der Waals and electrostatic interactions was analyzed, and it was found that the electrostatic interactions between aldehyde groups and water played a crucial role.

In this study, the effects of rapid synthesis of ZIF-90 in more environmentally friendly solvents and methods were investigated to explore the potential impact on structural and water adsorption properties. DMF was replaced by two alternative, less toxic dipolar solvents (protic methanol and/or aprotic acetone) and by water. The samples obtained have large specific surface areas, which is generally advantageous for the application of ZIF-90 in adsorption applications. In addition, the ZIF-90 with a specific surface area > 1200 m^2^/g was prepared within 5 min by microwave-assisted solvothermal synthesis. The advantage of using acetone solvent is not only evident in the structural properties of the obtained materials, but also in the fact that the final products do not require activation after synthesis (i.e., removal of captured solvent in the pores), which further reduces the cost of preparation. Furthermore, the ZIF-90s prepared in acetone and water solvent mixture, using both conventional and microwave-assisted approaches, show good hydrothermal stability, and the structures remain intact after 20 cycles of adsorption and desorption. To the best of the author’s knowledge, this is the first successful microwave synthesis of ZIF-90.

## 2. Results and Discussion

### 2.1. Synthesis

All ZIF-90 samples were prepared after 1.5 h at ambient pressure in an open glass beaker using water, acetone and methanol as a substitute for the hazardous solvent DMF. The samples were labelled with the abbreviations of the solvents used: AM—acetone and methanol, WAM—water, acetone and methanol, MW—methanol and water, and WA—water and acetone. The highest yield of 81% was obtained for the sample WA prepared in the water–acetone solution, whereas the yields of the other products were 56, 38, and 47 for the AM, WAM and MW samples, respectively. In addition, the sample MW was a predominantly water-based synthesized ZIF-90 with a V_water_/V_methanol_ volume ratio of 3. Due to the high yield and good structural properties, the acetone–water solvent mixture was also used for microwave-assisted solvothermal synthesis (named WA-Microwave). Using this approach, ZIF-90 was produced within 5 min at 80 °C with a yield of almost 80%.

### 2.2. Structural Properties of ZIF-90

**Figure 1:**
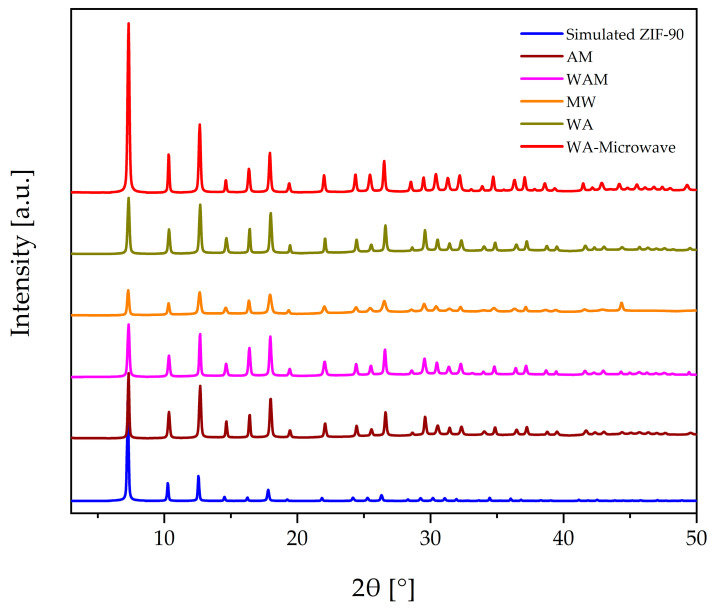
PXRD patterns of the ZIF-90 samples and the simulated ZIF-90.

Powder XRD patterns of the as-synthesized ZIF-90 samples (Figure 1) were compared with the simulated XRD pattern of ZIF-90, revealing the presence of a pure crystalline ZIF-90 phase. The diffraction maximum at 44 °2θ in sample MW belongs to the sample holder. According to the PXRD analysis, the structure of all samples was preserved after activation in vacuum at 150 °C. The highest diffraction intensities were determined for the WA sample prepared in microwave synthesis, while the lowest diffraction intensities can be observed for the MW sample (Figure 1), which also contains a small amount of amorphous phase. The higher relative intensity of the first diffraction peak in WA-Microwave sample, when compared to the other samples, is most probably due to the more pronounced pore-filling effect [34].

**Figure 2:**
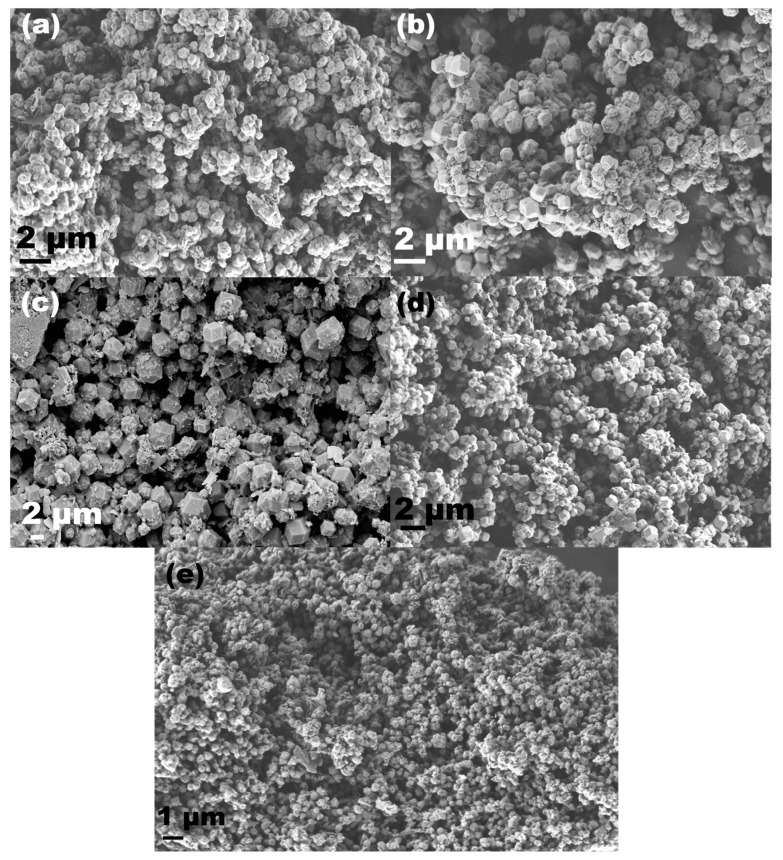
SEM images of (**a**) AM, (**b**) WAM, (**c**) MW, (**d**) WA, and (**e**) WA-Microwave.

SEM analysis was used to determine the morphology, phase purity, and particle size of the as-synthesized ZIF-90 materials (Figure 2). SEM images confirmed the phase purity of the samples prepared from the acetone-containing solvent mixtures as it can be seen from Figure 1. In addition, the particle sizes of the samples were not uniform and varied from 100 nm to 2 µm. Pure nanoparticles of 100 to 500 nm were formed when the samples were prepared from the solvent mixtures of acetone and methanol or water. Liu et al. [12] obtained pure ZIF-90 prisms (2–5 µm) after 24 h by solvothermal synthesis in methanol at elevated pressure and 70 °C. Larger hexagonal prisms (1–4 µm) were formed in methanol–water synthesis and the sample MW also contained some amorphous impurities, also detected by PXRD. On the other hand, when acetone was added to the mixture of methanol and/or water, prismatic nanoparticles were again obtained. Similar properties were observed in the sample WA-Microwave, where nanosized particles of 200 nm were formed. It can be clearly observed that the presence of acetone with another or both solvents in the reaction mixture led to the formation of pure and crystalline ZIF-90 nanoscale particles. The phenomenon of formation of different particle sizes in different solvents may be related to solvents’ physicochemical properties and consequent alterations in their interaction with the reactants [10,35,36,37]. Furthermore, while the XRD data indicate that the WA-Microwave sample contains the largest ordered crystalline domains, i.e., has the highest crystallinity, its particle sizes as determined by SEM are among the smallest of all samples. We expect that the observed particles in the other samples consist of smaller crystalline domains. Therefore, the Scherrer equation based on the collected XRD data was applied to further determine the particle size. This revealed a nanosized particle distribution of 40 to 100 nm, i.e., the calculated crystallite sizes were estimated to be 40 nm for the WA sample, 50 nm for the AM sample, 60 nm for the WAM sample, 70 nm for the MW sample and 100 nm for the WA-Microwave sample.

**Figure 3:**
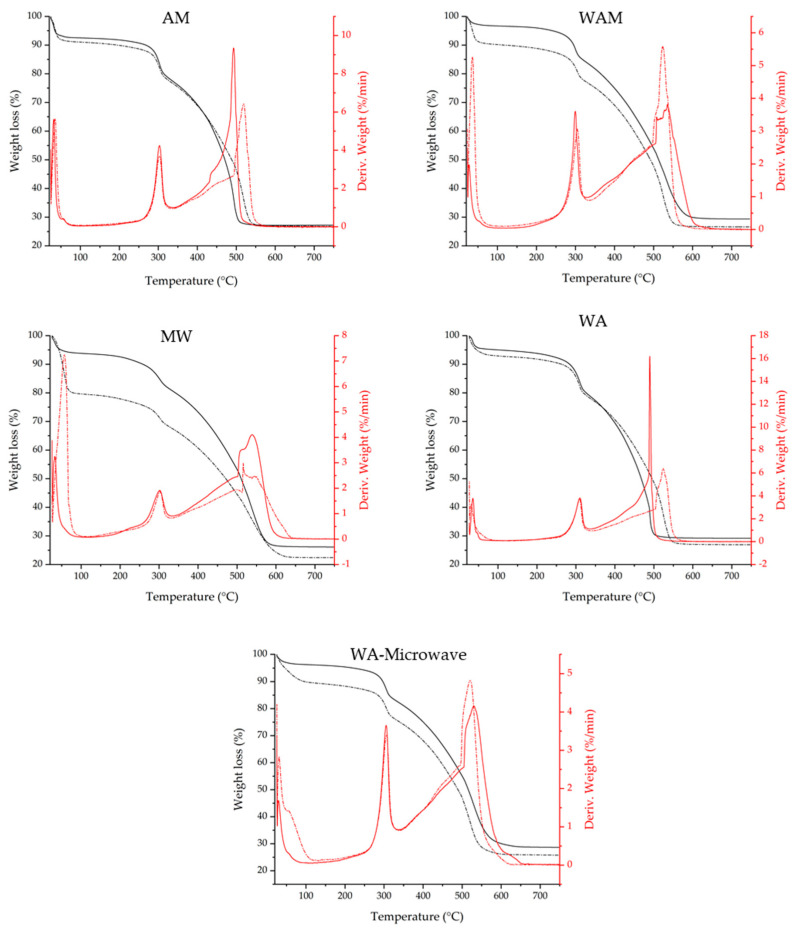
TG (black) and DTG (red) of the as-synthesized (dashed curve) and activated (solid curve) ZIF-90 samples.

To estimate the temperature at which the solvent can be removed from the structure and subsequently the optimal activation temperature of the material, thermogravimetric studies (Figure 3) were performed on the as-synthesized materials. In addition, the analysis was also performed on the activated samples to ensure that no solvent remained in the structure. After activation in a vacuum oven at 150 °C overnight, the materials exhibited a slight weight loss between 25 and 250 °C, which was due to the residual solvents on the material surface. At 300 °C and above, significant weight loss can be observed, which is attributed to the collapse of the ZIF-90 structure. TG analyses showed the difference in weight loss for the synthesized and activated samples. The difference in methanol-based ZIF-90s between the as-synthesized and activated samples indicated that some amount of solvent still remained in the material. The acetone-based samples showed little difference between the as-synthesized and the activated samples, indicating that acetone has evaporated from the structure, implying that activation of these samples is not needed.

**Figure 4:**
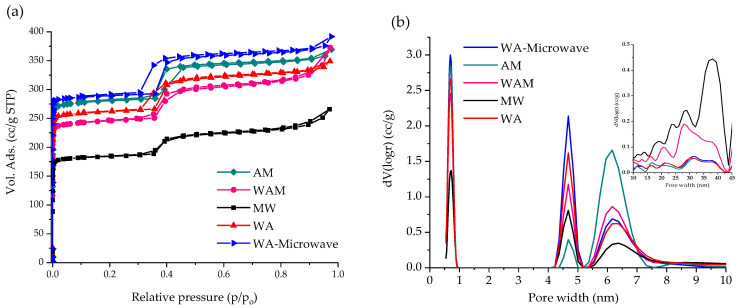
(**a**) Nitrogen physisorption isotherms for ZIF-90 samples at 77 K. (**b**) Pore size distributions for ZIF-90 samples (inlet: Pore size distribution from 10 to 45 nm).

Nitrogen physisorption isotherms for ZIF-90 samples are shown in Figure 4a. They show a mixture of Type I and Type IV isotherms for all ZIF-90 materials, indicating the presence of micropores and mesopores, while Figure 4b shows pore size distributions. Table 2 shows the structural parameters such as a specific surface area, micropore volume, mesopore volume, and total pore volume, determined from the nitrogen adsorption isotherms. Due to the nanoparticles, the samples prepared from the mixtures containing acetone–methanol or water have larger specific surface areas (>1000 m^2^/g) than the samples crystallized in methanol–water solution (752 m^2^/g) with particle sizes ranging from 1 to 4 µm. The sample AM formed in acetone–methanol solution at 60 °C has a similar specific surface area of 1166 m^2^/g as the ZIF-90 prepared in DMF-methanol solution at 70 °C [3]. Previous studies of ZIF-90 showed that the specific surface area of ZIF-90 is in the range of 514–1270 m^2^/g [3,7,8,10,12,13,38]. The order of specific surface areas of the samples prepared from acetone containing solutions is WA-microwave > AM > WA > WAM. These samples also have larger total pore and micropore volumes compared to the sample obtained from methanol–water solution. The specific surface areas are well correlated with particle size, i.e., samples with nanosized particles have larger specific surface areas. The ZIF-90 nanoparticles obtained via MAS exhibit the highest specific surface area (1215 m^2^/g). ZIF-90 with nanoparticles (100–500 nm) and specific surface areas ranging from 1028 to 1166 m^2^/g can be obtained only in acetone solutions, indicating that acetone promotes the nucleation process of nanoparticles [35].

### 2.3. Water Adsorption Properties

**Figure 5:**
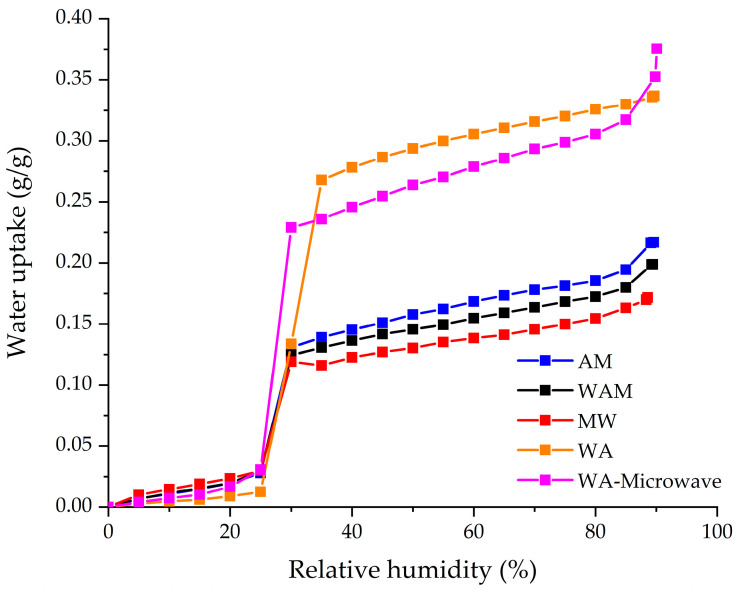
Water adsorption isotherms of ZIF-90 samples at 30 °C.

The influence of the synthesis method on the hydrophilicity of the ZIF-90 was investigated using water adsorption experiment. Figure 5 shows water isotherms of the ZIF-90 samples. All ZIF-90 samples exhibit a Type V water adsorption isotherm with hydrophobic–hydrophilic character (S-shape) with an initial low water uptake, followed by a sharp increase at 25% relative humidity (RH) and further slow increase till 90% relative humidity. The S-shape isotherm is beneficial for some heat transformation applications (heat pumps and chillers) since it enables a large uptake in a narrow relative humidity range [39]. The MW sample exhibits the lowest maximal water adsorption capacity starting with low water uptake in the range from 0–20% relative humidity, while at 25% relative humidity water uptake quickly increases from 0.03 g/g up to 0.12 g/g and then again slightly increases up to 0.17 g/g at 90% relative humidity. However, when acetone is added to the mixture of water and methanol, the maximal water uptake increases up to 0.20 g/g and 0.22 g/g for the WAM sample and the AM sample, respectively. When only acetone and water (WA) are used as solvents, the material shows improved hydrophilic properties by a significant increase in water uptake at 35% relative humidity up to 0.26 g/g and further increase up to 0.34 g/g at 90% relative humidity. A similar behavior can be observed in the WA-Microwave sample; however, the sharp increase is steeper but less intense (0.22 g/g), while the maximal water uptake is the highest among all the ZIF-90 samples (0.37 g/g). This indicates that the use of microwave-assisted synthesis can improve the maximal water uptake of the ZIF-90. It can be concluded that the combination of water–acetone solvents can significantly improve the hydrophilic properties of ZIF-90. The maximal water uptake of the WA sample is 55% higher than that of the MW sample, while the microwave synthesis additionally increases the maximal water uptake. It is known that during synthesis, different solvents and synthesis methods can lead to formation of different structural defects as possible adsorption sites [40].

**Figure 6:**
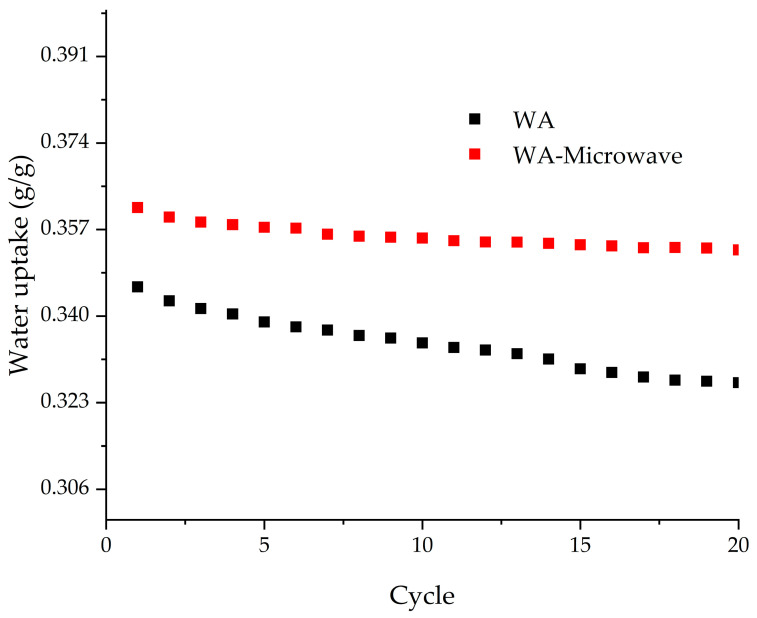
Cyclic stability of 20 cycles for the WA and WA-Microwave samples between 30 °C and 100 °C at 12.5 mbar.

ZIF-90s prepared in acetone–water mixture by conventional and microwave-assisted synthesis were further investigated to determine their hydrothermal stability between adsorption temperature of 30 °C and desorption temperature of 100 °C at water vapor pressure of 12.5 mbar. These conditions are usually used for low-temperature adsorption thermal battery [41,42] in applications such as building space heating. Prior to the cyclic tests, samples were degassed at 120 °C for 4 h. After 20 adsorption and desorption cycles the materials showed a slight change in water uptake. Figure 6 shows the water loading lift in the cycles of the materials, with the WA-Microwave sample showing better hydrothermal stability and higher water loading lift than the conventionally prepared ZIF-90. The water uptake after 20th cycle decreases by only 2% for the microwave synthesized ZIF-90, while the conventionally prepared sample shows 5% decrease in water uptake, indicating that microwave radiation during synthesis is favorable. However, both materials show good hydrothermal stability.

**Figure 7:**
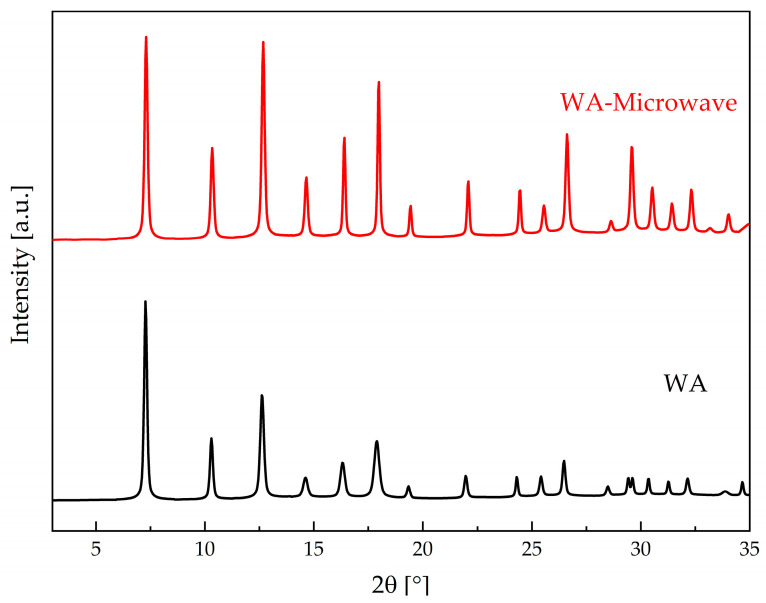
PXRD of ZIF-90 samples WA and WA-Microwave after 20 cycles.

To gain insight into the structural integrity after cyclic tests, PXRD of the used WA and WA-Microwave samples was performed. Figure 7 shows the PXRD of both materials after 20 cycles. The XRD patterns indicate the preservation of the structure, and the materials exhibit the resistance to structural deformation when exposed to cyclic water sorption tests.

## 3. Materials and Methods

### 3.1. Materials

1H-Imidazole-2-carbaldehyde (Hica, 97%) was purchased from Fluorochem (Had-field, UK). Zinc acetate dihydrate (ZnAc, 98%) was purchased from Sigma Aldrich (Darmstadt, Germany). Acetone (≥99.5%) was purchased from Honeywell Riedel-de Haën AG (Berlin, Germany) and methanol (MeOH, ≥99.8%) from Micro Polo (Maribor, Slovenia).

### 3.2. Conventional Solvothermal Synthesis of ZIF-90

ZIF-90 was synthesized using a previously modified procedure [3]. The research was oriented towards replacing DMF with other solvents (acetone, water and methanol) in various molar ratios. The used solvent mixtures for the ZIF-90 synthesis are listed in Table 3. Each ZIF-90 sample is named with an abbreviation for the used solvent in the synthesis, for example the name of the sample AM shows that the product was obtained from the solvent mixture of acetone and methanol, so A for acetone and M for methanol, while the sample WAM was formed from the mixture of water (W), acetone (A) and methanol (M).

ZnAc and HICA were dissolved in the particular solvent or solvent mixture in separate beakers. After the reactants were completely dissolved, the ZnAc solution was slowly added to the HICA solution. The resulting mixture was stirred for 1.5 h at 60 °C under ambient pressure. The resulting solutions were centrifuged at 6000 rpm for 6 min. The precipitate was washed twice with ethanol and dried overnight at 60 °C in an oven. All samples were then activated at the same conditions, i.e., under vacuum at 150 °C overnight to remove solvents or possible remains of solvents.

### 3.3. Microwave-Assisted Synthesis of ZIF-90

In order to obtain ZIF-90 according to the principles of green chemistry, the synthesis was carried out using a microwave-assisted solvothermal approach. Here, a mixture of acetone and water was used as solvent, since the crystallinity and yield are high in conventional solvothermal synthesis. HICA was dissolved in acetone in a Teflon bottle and stirred for 10 min. In the meantime, ZnAc was dissolved in water in a separate beaker. ZnAc solution was slowly added to the Teflon bottle and stirred for another 5 min. The Teflon bottle was then sealed in a microwave reactor and placed in a FlexiWave microwave (Milestone, Sorisole, Italy) and heated at 80 °C for 5 min. The precipitate was centrifuged at 6000 rpm for 6 min and washed twice with ethanol. The product was dried overnight at 60 °C and then activated overnight at 150 °C in vacuum. The sample is referred to as WA-Microwave.

### 3.4. Characterization

Powder X-ray diffraction (PXRD) was performed using a PANalytical X’Pert PRO diffractometer (Malvern Panalytical, Almelo, The Netherlands) with Cu Kα radiation (λ = 1.5418 Å). The 2θ range was 5–50° with a step size of 0.033°. The patterns were evaluated and the particle size calculated using the Scherrer equation using the X’Pert HighScore Plus program package. Nitrogen physisorption was performed using Autosorb iQ3 (Quantachrome Instruments, Boynton Beach, FL, USA). Isotherms were collected at 77 K. Prior to the measurement, the samples were degassed at 150 °C for 10 h in vacuum. The specific surface areas (S_BET_) were determined in the relative pressure range from 0.04 to 0.10 using the Brunauer–Emmett–Teller (BET) theory [43]. Total pore volume was estimated based on the amount of nitrogen adsorbed at a relative pressure of 0.95. The micropore volume of the samples was determined using the built-in algorithm based on the t-plot method and the pore size distributions were determined by the built-in algorithm based on DFT method. Scanning electron microscopic images (SEM) were acquired using a Zeiss Supra 35 VP microscope with a high electron voltage of 1.00 kV and an aperture size of 30.00 µm. Thermogravimetric analysis (TGA) was performed using TA Instruments Q5000IR. Measurements were made in continuous flow (25 mL/min air) from 25 °C to 750 °C, heating at a rate of 10 °C/min. Water adsorption studies and cyclic stability tests were carried out on IGAsorp-XT automated water sorption gravimetric analyzer (accuracy ± 0.1 µg) (Hiden Isochema Ltd., Warrington, UK) with the sequential procedure of water uptake measurements at 30 °C, under humid nitrogen gas flow (flow rate 250 mL/min). Prior to the adsorption experiments, samples were degassed at 120 °C for 4 h. The cyclic stability tests were performed with the sequential procedure of water uptake measurements at 30 °C, under humid nitrogen gas flow (250 mL/min and 80% relative humidity) for 4 h, followed by desorption at 100 °C in dry nitrogen flow for 4 h, which is typical for adsorption thermal battery applications [42]. A desorption temperature of 100 °C can be attained by solar thermal collectors, while adsorption temperature of 30 °C is sufficient for space heating applications. The water vapor pressure during desorption and adsorption of the samples was set to 12.3 mbar (a dew point temperature of 10 °C). The difference in the amount of adsorbed water at 35 °C and 100 °C at 12.5 mbar is the water loading lift of the sample in the cycle.

## 4. Conclusions

ZIF-90 was successfully synthesized with inexpensive solvent mixtures of acetone, water and methanol at 60 °C and ambient pressure in 90 min. Furthermore, when microwave-assisted synthesis was applied, the material was obtained at 80 °C in just five minutes. TG analysis of ZIF-90 synthesized in methanol–water solution showed solvent residues in the material, thus requiring activation of the material after synthesis. In contrast, when water and acetone were used as solvents, no additional activation was required, which reduced the cost of production. The solvent mixtures used in the preparation methods affected the maximum water adsorption capacity of ZIF-90, showing that ZIF-90 obtained from the acetone-water solution with microwave assistance achieved the highest water adsorption capacity (0.37 g/g), while the ZIF-90 formed in water–methanol solution reached the lowest water uptake (0.17 g/g). In addition, the ZIF-90 prepared from the acetone–water mixture had high specific surface area (>1100 m^2^/g), especially the microwave-synthesized ZIF-90 with >1200 m^2^/g. These materials also showed good hydrothermal stability; after 20 cycles under the adsorption thermal battery conditions, water uptake decreased by 5% and 2% for the conventionally and microwave-synthesized samples, respectively, and both ZIF-90 structures were preserved after 20 cycles. The green chemistry approach is visible not only from the aspect of the possibility of preparing materials in a laboratory microwave oven, but also by the fact that the acetone can be separated after the synthesis and reused in further syntheses.

## Figures and Tables

**Table 1:** ZIF-90 synthesis methods and the specific surface areas.

Solvents	Reaction Conditions	Synthesis Time	Specific Surface Area (m^2^/g)	Literature
Methanol	50 °C, autoclave	24 h	1150	[8]
DMF/Methanol	70 °C	30 min	1119	[3]
DMF	Room temperature	6 h	1387	[9]
GVL/Methanol	Room temperature	60 min	1136	[10]
Water/PVP/butanol	Room temperature	Few minutes	766	[13]
Methanol	85 °C, Autoclave	24 h	1461	[16]
Methanol/TEA	70 °C, reflux	4 min	1937	[17]
Water/PVP/butanol	Room temperature	5 min	553	[18]
DMF/TEA/Hexane	Room temperature	48 h	670	[19]
Water/PVP/butanol	30 °C	10 min	897	[20]

**Table 2:** Specific surface area and pore volumes of ZIF-90s.

ZIF-90	S_BET_ (m^2^/g)	V_micro_ (cm^3^/g)	V_meso_ (cm^3^/g)	V_total_ (cm^3^/g)
AM	1166	0.401	0.170	0.571
WAM	1018	0.349	0.227	0.576
MW	752	0.263	0.148	0.411
WA	1137	0.396	0.153	0.549
WA-Microwave	1215	0.423	0.183	0.606

S_BET_—specific surface area determined by the Brunauer–Emmett–Teller (BET) theory; V_micro_—micropore volume determined by t-plot method; V_meso_—mesopore volume; V_total_—total pore volume determined at 0.95 p/p_o._

**Table 3:** Molar ratio of reactants, solvents and the synthesis temperature.

Sample ZIF-90	ZnAc (mmol)	Hica (mmol)	Water [W] (mmol)	Methanol [M] (mmol)	Acetone [A] (mmol)	Synthesis Temperature (°C)
AM	0.33	1	-	62	34	60
WAM	0.33	1	69.5	20	17	60
WM	0.33	1	416	62	-	60
WA	0.33	1	55	-	135	60
WA-Microwave	0.3	1	317	-	267	80

## Data Availability

The data presented in this study is available on request from the corresponding author.
